# AutoSeqMan: batch assembly of contigs for Sanger sequences

**DOI:** 10.24272/j.issn.2095-8137.2018.027

**Published:** 2018-03-07

**Authors:** Jie-Qiong Jin, Yan-Bo Sun

**Affiliations:** 1 State Key Laboratory of Genetic Resources and Evolution, Kunming Institute of Zoology, Chinese Academy of Sciences, Kunming Yunnan 650223, China

**Keywords:** Batch processing, Sanger sequences, Contig assembly, SeqMan

## Abstract

With the wide application of DNA sequencing technology, DNA sequences are increasingly generated through the Sanger sequencing platform. SeqMan (in the LaserGene package) is an excellent program with an easy-to-use graphical user interface (GUI) employed to assemble Sanger sequences into contigs. However, with increasing data size, larger sample sets and more sequenced loci make contig assemble complicated due to the considerable number of manual operations required to run SeqMan. Here, we present the ‘autoSeqMan’ software program, which can automatedly assemble contigs using SeqMan scripting language. There are two main modules available, namely, ‘Classification’ and ‘Assembly’. Classification first undertakes preprocessing work, whereas Assembly generates a SeqMan script to consecutively assemble contigs for the classified files. Through comparison with manual operation, we showed that autoSeqMan saved substantial time in the preprocessing and assembly of Sanger sequences. We hope this tool will be useful for those with large sample sets to analyze, but with little programming experience. It is freely available at https://github.com/Sun-Yanbo/autoSeqMan.

## INTRODUCTION

DNA sequencing technology has experienced a revolutionary shift from automated Sanger sequencing (Sanger et al., 1977) to next-generation sequencing (NGS; reviewed by Shendure & Ji (2008) and Shendure et al. (2004)) and genome assembly. Although NGS has dominated due to its high throughput (Schuster, 2008), it is not suitable for many population studies due to high costs and other limiting factors. For example, errors are always introduced in final assembly and/or annotation results using NGS data (Bickhart et al., 2017), and thus variations detected in high-throughput analyses require validation by Sanger sequencing (Wall et al., 2014). Furthermore, for some present population genomic studies, error rates have been found to increase with increasing depth of coverage for Illumina data, and thus caution is needed when interpreting the results of next-generation sequencing-based association studies (Wall et al., 2014). As such, Sanger sequencing technology is still widely used in many research fields, including in evolutionary taxonomy based on short DNA sequences (Chen et al., 2017), evolutionary history study of wild animals (Yuan et al., 2016), biodiversity estimates and influencing factors (Zhou et al., 2017), and validation of mutations identified from high-throughput analyses (Sun et al., 2013).

Further, with the wide application of DNA sequencing technology, e.g. DNA barcoding, which uses short and standardized DNA sequences for individual identification of organisms (Hajibabaei et al., 2007; Savolainen et al., 2005), Sanger sequencing data are continuing to be accumulated among evolutionary taxonomists and others. Thus, batch manipulation of these Sanger sequences has become an important task before downstream analyses, especially for those who doesn’t have programming or bioinformatics background or experiments. Although several sequence manipulation packages for general purpose issues have been published previously, including MEGA (Kumar et al., 2016), EMBOSS (Rice et al., 2000), and FasParser (Sun, 2017), these packages are all based on assembled contigs (a consensus region of overlapping DNA segments) and no key consideration has been taken on the batch assembly of Sanger sequences.

**Figure 1:**
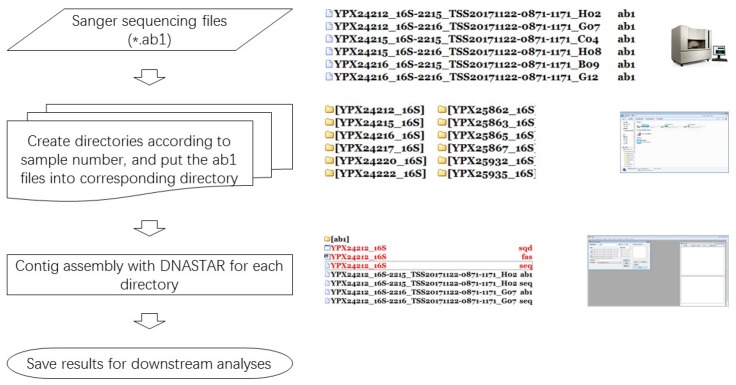
Overview of assembling tasks for Sanger sequences

SeqMan is a popular program in the LaserGene software package (DNAStar, Inc., Madison, WI, USA), which is used for assembling Sanger sequences into contigs and has been widely applied in a great number of studies. It can handle two to thousands of Sanger sequences at one time but requires a considerable number of manual operations (e.g., mouse actions, Figure 1) to run. Hence, it is complicated and time-expensive for those with large sets of samples to assemble. Fortunately, since the release of Version 7, SeqMan now provides a scripting language, including commands for opening, naming, saving, and closing projects, and a single script may be used to execute multiple assemblies consecutively without manual intervention.

Common tasks for assembling Sanger sequences include (1) classifying sequence files into corresponding folders (always completed manually) and (2) opening SeqMan to add the ab1 files that belong to a same sample and then assembling them (needing considerable mouse operations). The autoSeqMan program was designed based on these steps, with the ‘Classification’ and ‘Assembly’ functions corresponding to step 2 and 3, respectively.

Here, we developed a program called autoSeqMan, which provides a simple way to automatedly classify Sanger sequences and then consecutively assemble them on a personal computer. It is mainly designed for researchers with large sets of samples with one or more loci sequenced.

## IMPLEMENTATION AND REQUIREMENTS

autoSeqMan was developed into a standalone Windows desktop application (compiled and tested in Windows 7/10). It involves two modules, ‘Classification’ and ‘Assembly’, corresponding to steps 2 and 3 in Figure 1, respectively. Each module can handle multiple files and needs the user to select the directory either containing the raw Sanger sequence files (*.ab1 files) or containing the classified sub-folders created by ‘Classification’. Theoretically, there is no limit to the number of files that can be analyzed.

This tool requires that the sequence files be named in a specialized format, in which the sample ID should be present at the beginning of the file name. The Classification module will recognize the sample ID by the appropriate delimiter and then create sub-folders (see below). For convenience, autoSeqMan also provides a “Rename” tool to help users rename the ab1 files for the below analyses.

## CLASSIFICATION

This function is designed to automatedly create sub-folders according to the sample ID and/or sequenced locus. All downstream analyses are performed in the corresponding sub-folders, where all analyzed results are also saved. According to our laboratory experience, this is an efficient and convenient way to manage and query laboratory samples (Chen et al., 2017; Zhou et al., 2017).

**Figure 2:**
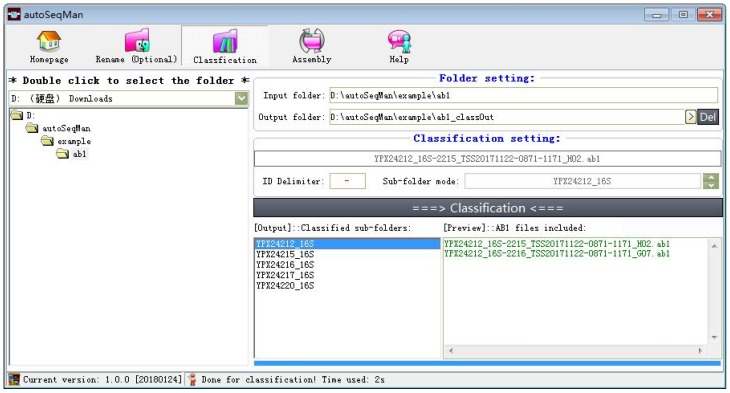
Overview of ‘Classification’ function in autoSeqMan

The only input is the directory name, which contains the raw ab1 files. There are several input prerequisites required for Classification performance. First, all files must be stored in a same directory. Second, all files must be named according to a certain pattern, i.e., “sample-locus-others”. For example, the file name “YPX24212_16S-2215_TSS20171122-0871-1171_H02.ab1” denotes that it is a DNA sequence of 16S and the sample number is “YPX24212”. The program will automatedly recognize the filename according to the user-specified delimiter and then create a sub-folder “YPX24212_16S” in the main output folder. The delimiter can be “-“, “_”, or other. After classification, the program will list all sub-folders, and the user can look at the files classified into each sub-folder by simply clicking the folder name (Figure 2).

To perform this function, user needs to tell autoSeqMan the full path of a directory which contains all raw ab1 files. The sample name should be present at the beginning of the file name, which will be recognized and extracted by the autoSeqMan according to the specified delimiter.

## ASSEMBLY

**Figure 3:**
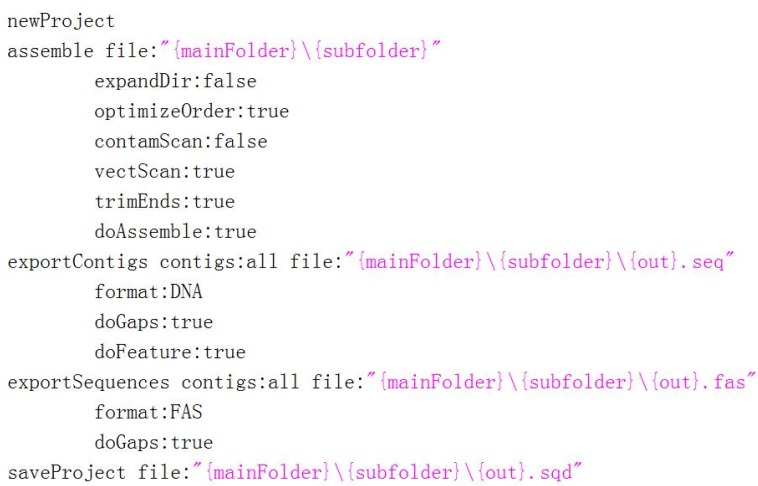
Default SeqMan script for assembling Sanger sequences

This function will automatedly assemble the classified sequence files. It will first read the list of classified sub-folders created by the ‘Classification’ function, and then generate a SeqMan script for consecutively assembling the sequences in each sub-folder. To perform this function, the user must first install the DNASTAR package (version 7 or higher), and then tell autoSeqMan the full path of the SeqMan program (which can be always recognized automatedly by autoSeqMan), after which the program will complete all assembly tasks and save the assembly results automatedly. The default script will generate all SQD, FAS, and SEQ results (Figure 3).

This script will be generated automated by autoSeqMan. There is no need for users to edit it. In default, all SQD, SEQ, and FAS outputs are generated.

## PERFORMANCE

The main aim of autoSeqMan is to save manual operation in preparing files and running the SeqMan program. To evaluate its performance, we applied this tool to our laboratory data (Chen et al., 2017; Zhou et al., 2017). In this test, one hundred samples were used, each of which had two ab1 files available. Results showed that the Classification operation created sub-folders (named sample ID as well as locus name if provided) and moved the appropriate files into the sub-folders within 8 s, substantially less than the time used for manual operation (about 1 h, as tested by our colleagues). Performance of the Assembly operation greatly depended on the running efficiency of SeqMan. In this test, the Assembly module required 64 s to consecutively assembly contigs for the classified sequences, also substantially less than the ~2 h required for manual operation, suggesting the significance of autoSeqMan in dealing with large date sets.

## LIMITATIONS

It is important to note that autoSeqMan does not undertake any filtration manipulation on the sequence data, even though poor-quality sequence ends are always present. Thus, after running autoSeqMan, users should undertake quality control measures of the final assembly with SeqMan. In addition, the output Fasta files will have very long IDs, which might introduce some errors in subsequent sequence analyses. If necessary, users can use the “Sort & Rename” function of FasParser (Sun, 2017) to shorten these IDs.
